# Dental Chemistry

**Published:** 1886-05

**Authors:** S. B. Palmer

**Affiliations:** Syracuse, N. Y.


### DENTAL CHEMISTRY.

BY S. B. PALMER, M. D. S., SYRACUSE, N. Y.

READ BEFORE THE FIFTH DISTRICT DENTAL SOCIETY OF THE STATE OF NEW YORK.

The time allowed to write this paper was as short as the subject is long, and it has required no little study to harmonize the two elements so as to present anything instructive.

For twelve years past it has been my duty in the State Board of Examiners to question candidates for the degree of M. D. S., upon this subject. The fact is revealed that some are well informed, while others know almost nothing of the science of chemistry. Therefore, a paper which would instruct and interest a chemist would be Greek to him who is ignorant. I will, therefore, ask the more fortunate to return with me for a few moments to the A B C of chemistry in the hope of encouraging private study and helping such as would gladly seek a practical knowledge of the subject.

Chemistry is defined as that branch of science which treats of the composition of substances, and of the changes which they undergo. It may be divided into four parts.

First.—Inorganic, which treats of minerals or inorganic substances.

Second.—Organic, which treats of substances that form the structure of organized beings and their products, whether animal or vegetable.

Third.—Practical or applied chemistry, which treats of the manufacture of the products of chemistry, their application to commercial purposes and of the conditions essential to their best use.

Fourth.—Pure chemistry, which treats of the element constitution of substances, of the laws of combination, and of the mutual reactions, and the relations therein involved.

It consequently explains modes and preparations of composition, and the process of decomposition and decay, and also the nature of the elements and of their compounds. Thus you see, Dental Chemistry, taken in the operative and mechanical department, involves each and all of the above named divisions, while many occupations might be limited to one or two branches.

It is hardly possible that dentists who are practicing without a knowledge of chemistry will become greatly proficient in their profession by individual study. Knowledge is power, no matter how obtained, and while many apply chemistry without any knowledge of the science, scientific knowledge would give a yet more extended field of practice. Our State laws make it obligatory upon those entering the profession within the State to know something of chemistry. We have those with us who formerly registered under the law without examination, and we presume without the advantage of a chemical education. I will endeavor to present the subject, both in language and by illustrations, so as to be understood, and will confine the teachings in chemistry to the department relating to our specialty. We must, however, refer to the rudiments in order to know what simple elements enter into combination in the formation of the dental organs.

Chemistry teaches that all matter of which we have any knowledge is composed of about sixty-five simple elements. From this small number, by various combinations and proportions, come the countless number of mineral, vegetable and animal forms, which comprise, surround and inhabit the globe. About three-fourths of this number of elements are classed as metals, and one-fourth non-metallic. In their simple or pure state little knowledge would be required to classify them accordingly.

The elements which enter into the dental organs probably do not exceed a dozen. In fact, it is claimed that thirteen only, called zoonic elements, taken from both classes, form all the structures in which life is manifested.

Leaving out proportions, Dental Chemistry teaches that human dentine consists of cartilage and vessels, phosphate of lime, fluoride of calcium, carbonate of lime, phosphate of magnesium and soda, with chloride of sodium. Those only who understand chemistry, know by this statement what simple elements enter into dentine or other portions of a tooth. The fault is not with the stinted analysis, but with those who cannot comprehend the constituents of the compounds given. The chemist would at once see by this analysis that dentine is composed of phosphorus, fluorine, calcium, carbon, magnesium and sodium.

Let us analyze carbonate of lime, a component part of the teeth, also of the salivary calculi, as well as limestone and marble. Take a piece of marble and apply heat as is done in lime-kilns, and we have two unlike substances—a gas known as carbonic acid gas, and quicklime. The gas when treated by heat, according to chemical instructions, is separated into carbon and oxygen. Send a current of electricity through the quicklime and there appear the white metal, calcium, and oxygen; with this all further attempts at division fails, and we say carbonate of lime is composed of carbon, calcium and oxygen, three simple elements. Thus the other compounds of tooth structure, chemically treated, at once reveal their constituents or simple elements. By reversing this order, compounds are formed from simple elements. This is called synthesis, going from simplicity to complexity, not alone to produce compounds which have been analyzed, but to form new ones not found in nature, like glass, soap, etc.

There appears to be no limit to the combinations and changes which may be produced by the union of even the thirteen elements which enter into living forms. In the characters representing the Morse Telegraphic Alphabet, we have a dot, a short and a long dash. By various arrangements of these symbols, all the letters and numerals are represented in a manner to give expression to the greater portion of our daily news. So with the elements. By the laws of affinity they unite in definite proportions, which may be determined with mathematical accuracy. What affinity really is we cannot tell; it is a force to be classed with cohesive gravitation, magnetism, etc. The combination of elements often produces unexpected results, and furnishes substances unlike their constituents, or even anything found in nature. Sand and alkali produce glass; alcohol and lime give chloroform; each wholly unlike its constituents. Again, sulphur and quicksilver unite to furnish vermilion, the coloring matter for red rubber. Elements combine in different proportions, giving very different compounds, yet each compound has its exact proportion. Nitrogen and oxygen, which are tasteless, when combined in equal proportions give nitrous oxide, which is sweet. One part nitrogen and five of oxygen give nitric acid, which is intensely sour.

Matter combines under influence of attraction or affinity. As already stated, dentine is composed of phosphorus, fluorine, calcium, carbon, magnesium and sodium, not taking into account oxygen, hydrogen, nitrogen, etc., found in the cartilage and vessels.

We must bear in mind that elements combine according to their affinity for the elements present. Whenever it occurs that a new element is introduced, with greater affinity for any one in the compound, the old relations are broken up and a new compound is formed. To illustrate; alcohol and camphor-gum will hold peaceable relations unless water be added. In that case, alcohol will unite with the water, and at once reject the camphor, which will appear in white flakes upon the surface. Thus it is easy to understand how acids affect tooth structure, and quite as easy to account for the presence of acids, when we consider that they are the result of fermentation. In the laboratory cavities cannot be produced by immersing teeth in acid, but when the experiments are conducted upon the principle that governs in the oral cavity, they furnish like results. The simplest experiment is accomplished by the electric current in a neutral or slightly alkaline solution, one pole of the battery furnishing acid to dissolve the dentine, while the lime is deposited at the other pole. Thus we may understand the procees of disintegration where food is confined between teeth or in cavities. Decomposition goes on undisturbed by the surrounding fluids.

In teeth which are filled with metallic fillings, unless certain conditions are observed, decomposition continues. Dryness is essential; that is, if the plug is imperfect, or the dentine too porous to prevent fluid circulation, thermal changes through the moisture excites sensibility, and in time destruction of the surrounding tissues follows. Amalgams which contain copper and silver, often by the oxides and sulphides arrest decay in frail teeth, but good amalgams, or rather such as we would choose to insert, as well as gold, need to be insulated, or so inserted that the porous dentine and the irregularities and angles of the cavity are filled with some indestructible varnish, the only method to my knowledge which will preserve an amalgam filling bright upon the inner or wall surfaces. One thing is certain; when amalgam remains bright next to dentine, there is no decomposition going on.

Dental Chemistry as a study is greatly undervalued. Organic chemistry should be taken into account in connection with histology and biology, by which more light might be gained respecting the growth and absorption of the dental organs.

Because bone is not an electrolyte in the laboratory, and for the reason that it cannot be deposited upon other articles by the electric current, like most metals, we ought not to declare the principle false in biology. It is not my intention to create a "New Departure" in the process of the growth or absorption of dentine. To my mind we have only to add to the principles now understood in physics what may be understood respecting vital force, to enable us to comprehend this subject which at present is but a theory, and not satisfactorily explained.

A knowledge of organic chemistry electro-biology and the electro-vital currents, would give to a comprehensive mind a definite idea of the principles and process of the growth of the dental organs, including exostosis and absorption, and not less the breaking down or building up of dentures according to constitutional changes. The key note is polarity; polarity of the elements furnished in the blood. It is well known that reversal of the current would take from the article being plated, and the metal thus taken would be deposited upon what before was the electrolyte, at the other pole of the battery.

Apply this principle to physiology, where other than metallic elements are influenced by electro-vital currents, and we may better comprehend the methods by which organs of living bodies undergo changes.

Thus it may be seen that Dental Chemistry, instead of being but one division of the four defined, embraces all, giving ample opportunity for study and investigation far beyond anything to be found in text-books, or even recorded in dental literature.

